# Tuberculosis of the patella masquerading as prepatellar bursitis

**DOI:** 10.1308/003588413X13511609955490

**Published:** 2013-01

**Authors:** S MacLean, S Kulkarni

**Affiliations:** Sandwell and West Birmingham Hospitals NHS Trust,UK

**Keywords:** Tuberculosis, Osteomyelitis, Prepatellar bursitis

## Abstract

Tuberculosis of bone is an uncommon entity in the Western world. We present a case of tuberculosis of the patella mimicking prepatellar bursitis in an otherwise fit and well woman of Bangladeshi origin. We believe tuberculosis of bone should form a differential diagnosis of the swollen knee in high risk patients.

## Case history

A 25-year-old woman of Bangladeshi origin presented with an insidious onset of swelling over her right knee. This had been present for three months following a fall. She had been treated previously with oral flucloxacillin by her general practitioner with no resolution of symptoms. Walking had become increasingly difficult. She had no other respiratory or systemic symptoms of note. She had no history of contact with infectious diseases and there had been no recent travel or family illness.

**Figure 1:**
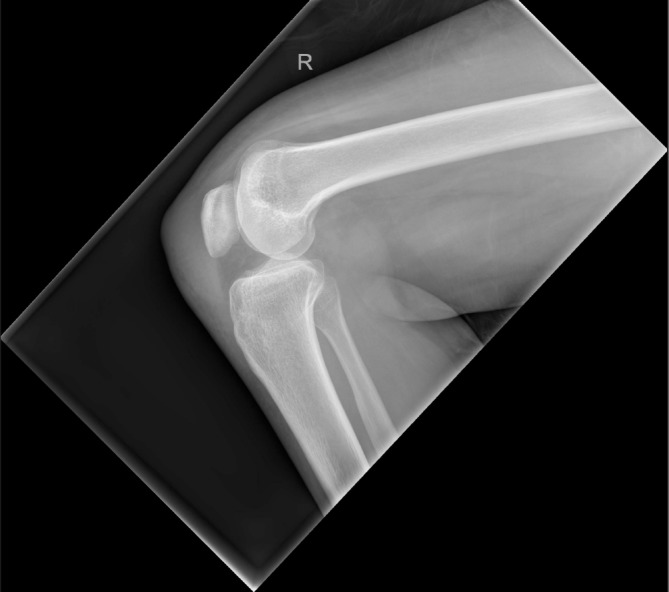
Plain lateral x-ray of the knee showing prepatellar soft tissue swelling; no obvious bony lesion

On examination, the patient had a large fluctuant prepatellar swelling with extensive surrounding erythema. There was a central punctum with a small amount of pus discharge. Range of movement was 0–110° but there was marked quadriceps wasting. The white cell count was normal, C-reactive protein (CRP) was 11mg/l and the erythrocyte sedimentation rate (ESR) was 30mm/hr. An x-ray showed a large prepatellar swelling but no obvious bony pathology (Fig 1).

The patient was diagnosed with an infected prepatellar bursitis, and taken to theatre for incision and drainage. At the time of surgery, 10ml of pus was drained and sent for microbiology, growing *Staphylococcus aureus*. No note was made of any patella abnormality at the time of surgery. After clinical improvement and a week of intravenous antibiotics she was discharged.

**Figure 2:**
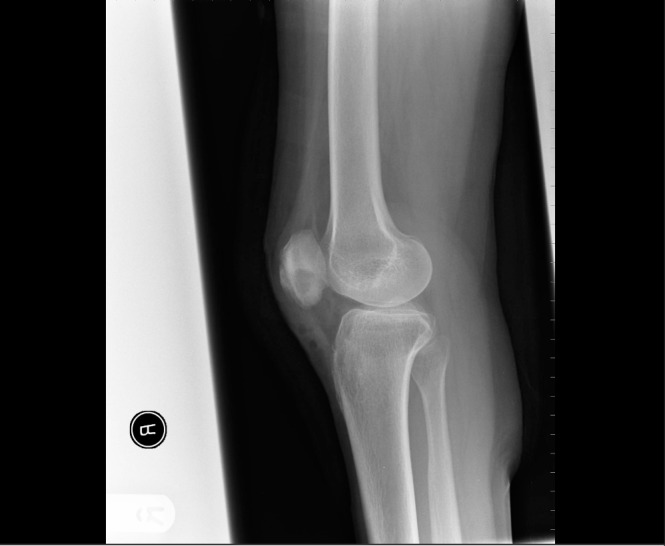
Lateral x-ray of the knee showing a focal lytic lesion in the patella with overlying soft tissue swelling and minor periosteal reaction; features consistent with a Brodie’s abscess

**Figure 3:**
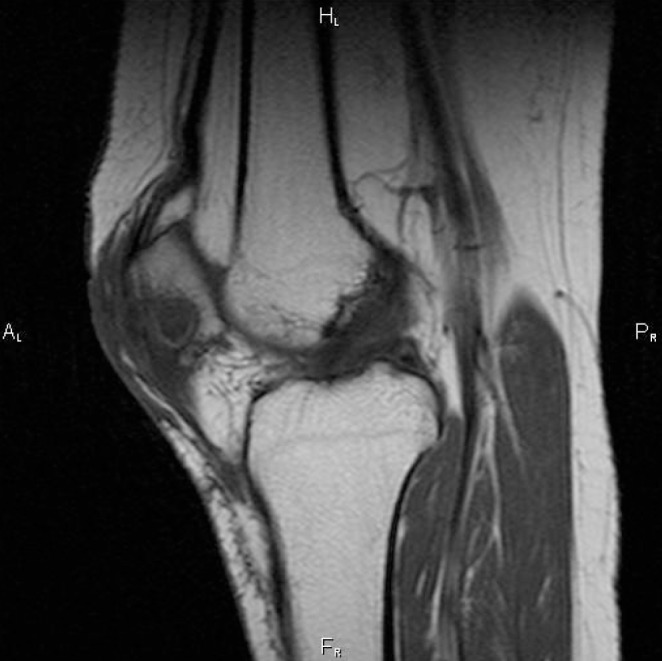
T1 weighted magnetic resonance imaging showing a 16mm × 12mm well defined lucency with central calcific densities, suggesting chronic osteomyelitis with sequestrum

A month later, the patient was admitted with a persistently painful discharging knee sinus. Respiratory examination revealed symmetrical breath sounds without crackles. There was no lymphadenopathy detectable on clinical examination. Chest x-ray showed no obvious pulmonary lesion. Further knee radiography showed a central nidus of necrotic bone in the patella (Fig 2). Magnetic resonance imaging (MRI) confirmed osteomyelitis (Fig 3). She was taken to theatre where a necrotic area of bone was found in the patella with granulomatous material surrounding it. There was no communication with the knee joint itself. A sinus excision and sequestrectomy was performed. Microbiology of the sequestered bone grew acid-fast bacilli, later confirmed as *Mycobacterium tuberculosis*.

The patient had an uneventful recovery from surgery, and was commenced on oral antituberculous medication of pyrazinamide and ethambutol by the respiratory team. No primary lung focus was found and no tuberculosis (TB) positive contacts were identified. She made a good clinical recovery with no recurrence of infection. At six months postoperatively, she has no pain in the knee, a full range of movement and return of quadriceps bulk.

## Discussion

TB of the patella is rare. Although the knee is the third most common site for skeletal TB after spine and hip involvement, the literature reports an incidence of patella TB of 0.09–0.15%.^1^ Although mostly reported in India, there have been other case reports from Europe^2–4^ but we found no case reported in the literature in a UK setting.

In our case, the patient was well systemically and gave no symptoms suggestive of chronic infection. Despite being from a country with a high prevalence of TB, she had no previous known contact with the disease. In TB, as in this case, CRP and ESR are often only mildly elevated or even normal. Suspicion of osteomyelitis was not expected owing to the delay in radiographic changes. X-ray features can vary. MRI is seen as the imaging of choice as it detects early bone marrow and soft tissue abnormalities although computed tomography has a role in detecting calcifications in abscesses. Ultimately, a tissue diagnosis is needed for diagnosis. Fluid aspirate may be negative or show normal skin flora unless specific TB microbiological tests are requested, as was shown in this case.

Treatment options depend on the extent and spread of osteomyelitis. Curettage can be used to remove sequestered bone and establish a tissue diagnosis. However, subsequent antituberculous therapy is seen as the mainstay of treatment.

Prepatellar bursitis is frequently associated with the minor trauma of repetitive kneeling, often occupational. Other common causes include infection, inflammatory causes (eg rheumatoid arthritis) and crystal deposition diseases (eg gout). Prepatellar bursitis as a sequela to patella osteomyelitis is not uncommon and should be considered on acute presentation. We could find one case of *M tuberculosis* presenting as prepatellar bursitis in the literature.^5^ Other rare causes of prepatellar bursitis should be considered such as sarcoidosis, arteriovenous malformation and pathology mimicking prepatellar bursitis (eg neoplasia or Morel-Lavallée lesions).

## Conclusions

The incidence of TB in the developed world remains high owing to immunosuppression (particularly with human immunodeficiency virus infection), the emergence of multidrug resistant TB and newly settled immigrants to Europe from highly endemic areas. We believe that all surgeons assessing patients from TB endemic regions presenting with an acute joint swelling should be suspicious of TB as a differential diagnosis. Prepatellar bursitis is often used as a blanket term to steer management in the emergency setting. In high risk patients with prepatellar bursitis, when x-rays and blood tests are unremarkable, further diagnostic tests and imaging are required, to aid diagnosis and management.
